# Radiofrequency echographic multi spectrometry (REMS) in the diagnosis and management of osteoporosis: state of the art

**DOI:** 10.1007/s40520-024-02784-w

**Published:** 2024-06-21

**Authors:** Nicholas R Fuggle, Jean-Yves Reginster, Nasser Al-Daghri, Olivier Bruyere, Nansa Burlet, Claudia Campusano, Cyrus Cooper, Adolfo Diez Perez, Philippe Halbout, Tullio Ghi, Jean-Marc Kaufman, Andreas Kurt, Radmila Matijevic, Regis P Radermecker, Sansin Tuzun, Nicola Veronese, Rene Rizzoli, Nicholas C Harvey, Maria Luisa Brandi, Maria-Luisa Brandi

**Affiliations:** 1 MRC Lifecourse Epidemiology Centre, University of Southampton, Southampton, UK; 2 Faculty of Medicine, Department of Public Health Sciences, Research Unit in Public Health, Epidemiology and Health Economics (URSAPES), University of Liège, Liege, Belgium; 3 The European Society for Clinical and Economic Aspects of Osteoporosis, Osteoarthritis and Musculoskeletal Diseases (ESCEO), Liege, Belgium; 4 Protein Research Chair, Biochemistry Department, College of Science, King Saud University, Riyadh, Kingdom of Saudi Arabia; 5 Chair for Biomarkers of Chronic Diseases, Biochemistry Department, College of Science, King Saud University, Riyadh, Kingdom of Saudi Arabia; 6 Faculty of Medicine, Clinica Universidad de los Andes, Universidad de los Andes, Santiago, Chile; 7 NIHR Southampton Biomedical Research Centre, University of Southampton, Southampton, UK; 8 NIHR Oxford Biomedical Research Centre, University of Oxford, Oxford, UK; 9 Department of Internal Medicine, Hospital del Mar-IMIM-UAB, CIBERFES, Institute Carlos III, Barcelona, Barcelona, Spain; 10 The International Osteoporosis Foundation (IOF), Nyon, Switzerland; 11 Department of Medicine and Surgery, University of Parma, Parma, Italy; 12 Department of Endocrinology, Ghent University Hospital, Ghent, Belgium; 13 Department of Orthopaedic and Trauma Surgery, Community Clinics Middle Rhine, Campus Kemperhof, Koblenz, Germany; 14 Faculty of Medicine, Clinic for Orthopedic Surgery and Traumatology, University of Novi Sad, Clinical Center of Vojvodina, Novi Sad, Serbia; 15 Department of Diabetes, Nutrition and Metabolic Disorders, Clinical Pharmacology, University of Liege, CHU de Liège, Liège, Belgium; 16 Department of Physical Medicine and Rehabilitation, Cerrahpaşa School of Medicine, Istanbul University- Cerrahpaşa, Istanbul, Turkey; 17 Department of Internal Medicine, Geriatrics Section, University of Palermo, Palermo, Italy; 18 Division of Bone Diseases, Faculty of Medicine, Geneva University Hospitals, Geneva, Switzerland; 19 Metabolic Bone Diseases Unit, Department of Surgery and Translational Medicine, University of Florence, Florence, Italy

**Keywords:** Radiofrequency echographic multi spectrometry (REMS), Fracture, Osteoporosis, Screening, Diagnosis

## Abstract

Radiofrequency Echographic Multi Spectrometry (REMS) is a radiation-free, portable technology, which can be used for the assessment and monitoring of osteoporosis at the lumbar spine and femoral neck and may facilitate wider access to axial BMD measurement compared with standard dual-energy x-ray absorptiometry (DXA).

There is a growing literature demonstrating a strong correlation between DXA and REMS measures of BMD and further work supporting 5-year prediction of fracture using the REMS Fragility Score, which provides a measure of bone quality (in addition to the quantitative measure of BMD).

The non-ionising radiation emitted by REMS allows it to be used in previously underserved populations including pregnant women and children and may facilitate more frequent measurement of BMD.

The portability of the device means that it can be deployed to measure BMD for frail patients at the bedside (avoiding the complications in transfer and positioning which can occur with DXA), in primary care, the emergency department, low-resource settings and even at home.

The current evidence base supports the technology as a useful tool in the management of osteoporosis as an alternative to DXA.

**Supplementary Information:**

The online version contains supplementary material available at 10.1007/s40520-024-02784-w.

## Introduction

Osteoporosis is a disease of bone characterised by loss of bone mass and microarchitectural deterioration associated with an increased risk of fragility fracture. It is highly prevalent, affecting over 200 million people worldwide, in many populations, 1 in 2 women and 1 in 5 men over the age of 50 years experiencing a fragility fracture in remaining lifetimes [1–4].

Osteoporosis is a silent disease until the moment of fracture, and thus assessment of fracture risk and bone mineral density plays a vital role in diagnosis and therapeutic decision-making. Dual-energy X-ray Absorptiometry (DXA) assessment of bone mineral density (BMD) at the axial reference sites (lumbar vertebrae and femoral neck) is the basis of the World Health Organisation’s operational definition of osteoporosis, with the femoral neck more recently espoused as the reference site for epidemiological purposes and fracture risk assessment [5, 6]. Other modalities are also used, some limited to a research setting, including Quantitative Computed Tomography (QCT), peripheral QCT (pQCT, which measures bone microarchitecture at peripheral sites such as the radius and tibia), High-Resolution pQCT (HR-pQCT), Quantitative Ultrasound (QUS) and Magnetic Resonance Imaging (MRI) [5, 7]. All the above modalities have various limitations which individually include ionising radiation (CT techniques), low portability, limited access/availability in clinical practice (particularly for those only used in a research setting: pQCT and MRI), prolonged acquisition times (MRI) and technical/operator-dependent variation (QUS) [7].

Radiofrequency Echographic Multi Spectrometry (REMS) is a radiation-free, portable technology which can be used for the assessment of osteoporosis at the central (rather than peripheral) regions including lumbar vertebrae and femoral neck and presents potential advantages over DXA and other modalities. This narrative review will explain how REMS works and how it can be used for the assessment of fracture risk, examine the deployment of REMS in specific populations, including pregnancy, postmenopausal women, chronic disorders of the skeleton and secondary fracture prevention, and provide recommendations for the use of REMS in clinical practice.

## How does REMS work?

**Fig. 1:**
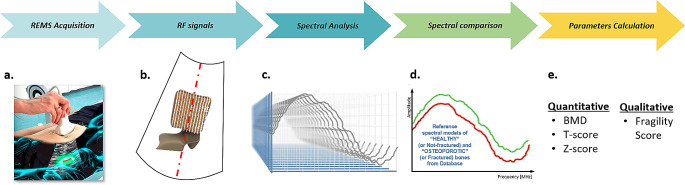
REMS basic principles: **(a)** Lumbar spine REMS scan. **(b)** Simultaneous acquisition of the native raw unfiltered signals of several scan lines considering all available tissue information. **(c)** Dedicated spectral processing of the acquired signals. **(d)** Comparison between ROI spectra specific for the patient and those of the reference model spectra of healthy and pathological patients, matched by age, sex, BMI and anatomical site. **(e)** Calculation of quantitative and qualitative parameters

REMS allows bone health status assessment and fracture risk prediction by means of a rapid ultrasound scan of reference axial sites (at the lumbar spine and proximal femur). It uses a transducer to emit ultrasound at the target site, and the resultant back-scattered waveforms are then captured by the receiver and undergo B-mode image reconstruction of the region of interest. Radiofrequency signal analysis is automatically performed to allow the identification of bone interfaces and regions of interest (ROIs), and to determine the status of internal bone microarchitecture. Via this method, key elements of anatomy, including each individual vertebral body, the femoral neck, femoral head and greater trochanter, can be identified and the corresponding BMD level together with the bone quality can be assessed and quantified [8, 9]. The basic principles of REMS are schematically summarised in Fig. 1.

Automated identification of target bone structures is achieved via a number of image processing steps applied to each image frame including the rearrangement of image data features in rectangular matrices, brightness masking, contrast enhancement, image smoothing, histogram equalisation, thresholding and morphologic evaluations [9].

An advantage of REMS over DXA is that artefacts, caused by calcifications, osteophytes, vertebral fractures, metal structures, etc. are automatically accounted for, potentially leading to more accurate measures of BMD, as recently documented by studies in both Caucasian and Japanese subjects [10–12]. The measurements can be performed quickly at both the femoral neck (40 s) and lumbar spine (80 s).

## What does REMS measure?

**Fig. 2:**
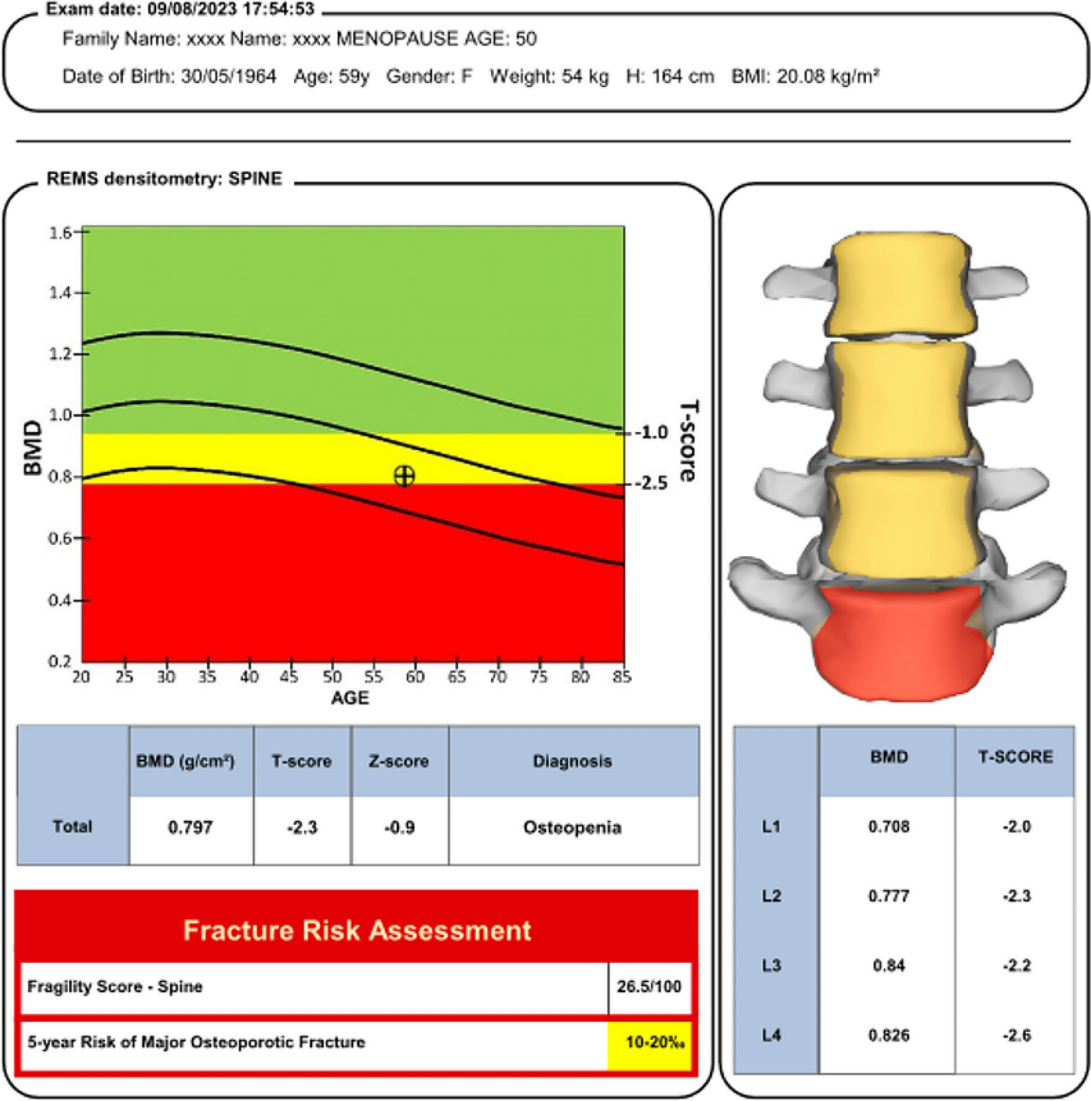
Example of REMS diagnostic report for a lumbar spine scan

The radiofrequency signal undergoes spectral analysis and the resultant waveform can be compared to data from reference populations (including ‘normal’ and ‘osteoporotic’ subjects) and quantitative parameters are calculated such as BMD values as well as T-scores and Z-scores (see Fig. 2), comparable with those outputs from DXA. BMD values are provided for each lumbar vertebra, the femoral neck, total hip and greater trochanter. The measure of REMS BMD is based on spectral models originally derived from a reference population that underwent also DXA to define osteoporosis, which were double-checked by experienced operators to avoid possible errors (including wrong patient positioning, inaccurate data analysis, presence of artifacts, etc.) that could provide unreliable BMD values [7, 13]. The methodology adopted to derive the reference population was described in detail in a previous paper [9] and essentially consists of population-based data that were gathered and grouped into 5-yearly intervals based on subject age, including 100 subjects for each considered age group.

Fragility score is a REMS measure of skeletal fragility (via bone microarchitecture and independent of BMD) at the spine and femoral neck and ranges from 0 (normal) to 100 (maximum fragility of the bone structure). It is derived from the proportion of scan lines whose spectra are more correlated with a “fragile” (i.e. fractured) bone spectral model than with a normal bone spectral model. The Fragility Score metric is used to generate measures of Fracture Risk over a five-year time horizon, using models derived from a proprietary database including datasets acquired on both fractured and non-fractured subjects [14, 15]. The diagnostic performance of Fragility Score in predicting incident fragility fractures at 5 years has been validated in comparison to BMD T-scores measured by both DXA and REMS, therefore considering the actual occurrence of major or hip fragility fractures as the reference “gold standard” [15].

## REMS BMD diagnostic performance

The accuracy and precision of REMS has been first examined in an Italian, multi-centre study (including 1914 women aged 51–70 years) [8]. REMS performed well at discriminating BMD defined osteoporosis, with sensitivity of 91.5% at the femoral neck and 91.7% at the lumbar spine and specificities of 91.8% at the femoral neck and 92.0% at the lumbar spine [16]. Discrimination accuracy was satisfactory when tested without any tolerance for deviation (agreement rates of 88.2% at the femoral neck and 88.8% at the lumbar spine) and became excellent with a tolerance of a 0.3 T-score (98.0% at the femoral neck and 97.4% at the lumbar spine). The precision of REMS indicated low intra-operator variability (measured via root mean square coefficient of variation (RMS-CV) at 0.32% and 0.38% for the femoral neck and lumbar spine respectively), which means a high level of test precision. The inter-operator variability (also RMS-CV) was just slightly higher at 0.48% for the femoral neck and 0.54% for the lumbar spine. When comparing the REMS BMD T-scores with DXA there was strong correlation at both the lumbar spine (*r* = 0.94, *p* < 0.001) and femoral neck (*r* = 0.93, *p* < 0.001)^8^.

Thanks to the reported precision and repeatability values, REMS BMD measurements can be effectively used for short-term therapeutic monitoring, overcoming the limitation of the other densitometric techniques which typically require at least 1 year between two scans. This is supported by the first scientific evidences demonstrating the feasibility of 6-month follow-ups using REMS to quantify the BMD decrease due to aromatase inhibitors-based treatment in breast cancer patients, and to assess the BMD recovery following denosumab administration [17, 18]. Analogous results in terms of feasibility and effectiveness are also reasonably envisaged from REMS employment for the monitoring of anabolic therapy effects.

In order to examine the discriminative performance of REMS more broadly, a wider, European multi-centre study was performed (4307 women from UK, Belgium, Italy and Spain, aged 30–90 years). High sensitivity for discrimination of DXA BMD osteoporosis was observed at the femoral neck (90.4%) and lumbar spine (90.9%), together with high specificities of 95.5% and 95.1% at the femoral neck and lumbar spine respectively [19]. High correlation was recorded between REMS BMD and DXA BMD (r_Pearson_ = 0.93 femoral neck, r_Pearson_ = 0.94 lumbar spine) and areas under the receiver operator characteristic curve (AUC) for classification of those who had and had not sustained a fracture were 0.683 at the femoral neck and 0.640 at the spine, indicating a discriminative performance for this population higher than DXA for the same patients (corresponding AUC values for DXA were 0.631 and 0.603, respectively) [19]. It should be noted that a substantial proportion of scans were excluded from these analyses (DXA: 8.0% femoral neck, 9.6% lumbar spine, REMS: 7.6% femoral neck, 8.8% lumbar spine). However, the same paper also reports the real-world data (“unchecked real-life scenario”), which substantially confirm the above findings in what might be expected in the context of an average clinical osteoporosis service: *r* = 0.88 for femoral neck, *r* = 0.90 for lumbar spine, sensitivity of 85.5% for femoral neck and 89.0% for lumbar spine, specificity of 94.5% for femoral neck and 94.3% for lumbar spine [19].

Further examination of the relationship between REMS and DXA was conducted via a study at the femoral neck examination in two osteoporotic populations, one with primary osteoporosis and one with disuse-related osteoporosis, for a total of 175 patients. The diagnostic concordance was 63% (Cohen’s kappa = 0.31) in patients with primary osteoporosis and 13% (Cohen’s kappa = -0.04) in those with disuse-related osteoporosis [20]. In the primary osteoporosis group (*n* = 140) there was no significant difference between femoral neck and total femur BMD measures (mean difference between REMS and DXA: -0.015 g/cm^2^ and − 0.004 g/cm^2^, respectively), confirming a good agreement between REMS and DXA in primary osteoporosis patients; however, there was a significant difference in the disuse-related osteoporosis group (*n* = 35, mean differences of 0.136 g/cm^2^ and 0.236 g/cm^2^ respectively) demonstrating poor diagnostic concordance but only in this smaller population [20]. The same study reported a statistically significant ability of Fragility Score to discriminate between fractured and non-fractured patients for both the considered groups (primary osteoporosis and disuse-related osteoporosis) and an excellent test-retest reproducibility of REMS measurements (measured Interclass Correlation (ICC) values between two consecutive REMS measurements were in the range 0.976–0.998).

When considering the precision and repeatability of the Fragility Score, the intra-operator variability is minimal (RMS-CV = 0.49% for lumbar spine and RMS-CV = 0.43% for femoral neck), as is the inter-operator variability (RMS-CV = 0.73% for lumbar spine and RMS-CV = 0.64% for femoral neck) [15]. This is thought to be due to the automated selection of the region of interest and has been borne out in a recent review of REMS with excellent agreement and accuracy reported [7].

The performance of REMS was further examined in a “real-life” setting of 343 women aged 30–80 years in Brazil [21] including different ethnicities (Asian, Caucasian, African descendent and “Miscegenated”). Although a quite large number of scans were excluded because of poor quality acquisitions with both methods or other technical reasons (41 lumbar spine DXA, 30 hip DXA, 67 lumbar spine REMS and 63 hip REMS), indicating that probably the operators had not actually completed their learning curves, the AUC for REMS predicting DXA-defined osteoporosis was very high (AUC = 0.97).

A further use of REMS technology in clinical practice was also reported in a representative cohort of 455 Mexican women of Hispanic ethnicity aged over 40 years covering a very broad BMI range (16.8–48.3 kg/m^2^), with a high proportion of pre-obese and obese subjects (72.1%). REMS scans were feasible on the whole cohort of enrolled subjects, independent of BMI and showing diagnostic classifications in line with the expected prevalence of osteoporosis/osteopenia in the considered population [22].

## Fracture prediction

In order for REMS to be used effectively in clinical practice it is not sufficient for it to correlate with DXA BMD, as shown by the papers discussed in the previous section, but its ability to predict incident fractures.

This has been examined in a population of 1516 Caucasian women aged 30–90 years [23], who were recruited, underwent REMS and DXA BMD assessment at axial sites and were followed-up for a mean of 3.7 years. Fractures occurred in 14.0% of women and stratified analysis into age-matched fracture (*n* = 175) and non-fracture (*n* = 350) groups showed statistically significant differences in BMD in these two groups (for both DXA and REMS) [23]. At the lumbar spine, and using a threshold of T-score = -2.5 to separate osteoporotic and non-osteoporotic patients, REMS identified fracture patients with a sensitivity of 65.1% and specificity of 57.7% (Odds Ratio (OR) = 2.6, 95%CI: 1.77–3.76, *p* < 0.001), with DXA demonstrating lower sensitivity (57.1%) and specificity (56.3%) (OR = 1.7, 95%CI: 1.20–2.51, *p* < 0.01) [23]. At the femoral neck, REMS sensitivity and specificity were 40.2% and 79.9%, (OR = 2.81, 95%CI: 1.80–4.39, *p* < 0.001) with similar sensitivity and specificity using DXA of 42.3% and 79.3%, respectively (OR = 2.68, 95%CI: 1.71–4.21, *p* < 0.001) [23]. A significantly better AUC for fracture discrimination was observed for REMS BMD T-score (AUC = 0.66) compared to DXA BMD T-score (AUC = 0.61) at the lumbar spine (*p* < 0.001), whereas the AUCs did not differ significantly at the femoral neck (AUC = 0.64 for REMS, AUC = 0.65 for DXA, *p* = 0.38) [23].

The use of Fragility Score to predict fracture risk has been tested in a prospective, 5-year follow-up study in 1989 Caucasian men and women [15]. The diagnostic performance to predict future major osteoporotic fractures (over the next 5 years) was good in both women (AUC = 0.811) and men (AUC = 0.780) (AUC = 0.809 in men and AUC = 0.780 in women after adjustment for age and BMI) [15]. For the specific performance of 5-year hip fracture prediction the performance was again good for both men (AUC = 0.809) and women (AUC = 0.780) (attenuated to AUC = 0.758 for men and AUC = 0.735 for women after adjustment for age and BMI) [15]. Overall, the Fragility Score performance in fracture prediction for both femur and spine, in either women or men, was superior to both REMS and DXA T-scores for BMD, which recorded AUCs ranging from 0.472 to 0.709 [15].

In summary, REMS BMD and DXA BMD seem to be similarly predictive of incident fracture and REMS Fragility Score may provide enhanced future fracture prediction through the additional information accrued via this technique. Future work in the field of fracture prediction should include a focus on non-Caucasian ethnicities.

## REMS assessment in pregnancy

The absence of ionising radiation in the deployment of REMS (in contrast to DXA) allows it to be used in pregnancy. This has opened opportunities to measure skeletal changes related to osteoporosis or vitamin D deficiency (and osteomalacia) during gestation. Whilst routine obstetric clinical opportunities are scarce, for those at high risk of musculoskeletal sequelae or experience rare conditions such as Pregnancy and Lactation Associated Osteoporosis or Transient Pregnancy associated Osteoporosis of the Hip, REMS may facilitate safe clinical assessment [24–26].

The homeostasis of bone metabolism can be affected by pregnancy with increased osteoblastic activity with higher levels of oestrogen counteracted by increased osteoclastic activity as skeletal calcium is mobilised to meet the foetal demand, with a net movement of calcium across the placenta towards the foetus. This increased osteoclastic activity is associated with clinical sequelae including increased bone fragility, increased fracture risk, increased joint pain and the development of Transient Pregnancy associated Osteoporosis of the Hip (TOH) [24, 25]. Therapeutic steroid usage, to prepare the foetal lungs for premature delivery, and reduced physical activity during pregnancy can also add to the demineralisation of bone.

Previous studies, performing DXA before (preconception) and after (postpartum) pregnancy, have demonstrated a 3% decrease in femoral neck BMD across pregnancy [27] and one study which performed a mid-pregnancy DXA at 12–20 weeks demonstrated a mean 0.01 g/cm^2^ loss of femoral neck BMD between the first scan (at 12–20 weeks) and the second scan (postpartum) [28]. Studies using QUS, which shares the advantage of emitting non-ionising radiation, are limited to the analysis of peripheral anatomical sites (including the calcaneum) which are more trabecular and so may be more liable to change than more cortical sites in the axial skeleton, such as the femoral neck [7]. However, studies using QUS demonstrated a net reduction in maternal BMD across the pregnancy period [29–31]. Whilst changes during pregnancy appear modest, there appears to be more bone loss during lactation, but with losses recovered over time [32, 33]. 

A case-control study comparing REMS BMD at the femoral neck of 78 women during an uncomplicated pregnancy to a non-pregnant control population (*n* = 78, matched on the basis of age, BMI, ethnicity and parity and recruited from a REMS database) showed that the pregnant women had, on average, a 8.6% lower BMD compared to the controls [34]. Further work has been performed to monitor changes in REMS BMD between the 1st and 3rd trimester with a mean reduction in BMD of 2% during this period, though no predictors for loss were identified [35].

Overall, REMS may represent a safe and effective approach to assess BMD during pregnancy and to trigger targeted interventions (e.g., Vitamin D supplementation), as well as to monitor bone health during the post-partum period and lactation.

## Chronic disorders affecting the skeleton

REMS has been investigated in a variety of physiological, ageing and disease states a study in Bulgaria compared REMS BMD in premenopausal women to postmenopausal women and found a significantly lower REMS BMD in the latter at both lumbar spine (premenopausal BMD = 0.942, postmenopausal BMD = 0.820, *p* < 0.001) and femoral neck (premenopausal BMD = 0.713, postmenopausal BMD = 0.646, *p* = 0.01) [36].

Patients with diabetes are known to be at increased risk of fracture despite a paucity of changes in DXA BMD. For this reason, Caffarelli and colleagues examined REMS and DXA BMD in a cohort of 90 Caucasian patients with Type 2 diabetes mellitus (aged 50–80 years). They found that BMD T-scores at the lumbar spine, femoral neck and total hip were significantly lower with REMS than with DXA, and thus that REMS led to 47% of the population being diagnosed with osteoporosis (compared to 28% with DXA). There was also a significant difference in lumbar spine BMD between those who had and had not sustained a previous fragility fracture (22 participants) using REMS (whereas this difference was not observed when considering DXA BMD), indicating a possible advantage of REMS employment for the identification of diabetic patients with increased fracture risk. Whilst these findings are intriguing, it is not known how they might actually impact on fracture risk prediction on these specific patients before a dedicated longitudinal study is performed. In fact, it should be recognised that diabetes-related bone diseases are not simply osteoporosis but also glycation of non-collagenous proteins and other skeletal causes of bone fragility which may not match the osteoporosis phenotypes [37]. This may have implications for the extent to which BMD measurements in general can be utilised in monitoring bone health in diabetic patients beyond the realm of osteoporosis, at the same time opening further interesting perspectives for possible dedicated applications of independent parameters such as the Fragility Score.

REMS has also been used to replicate DXA findings demonstrating lower REMS BMD in a population of rheumatoid arthritis patients (*n* = 91) compared to healthy controls (*n* = 116) [38]. In a further study REMS T-score for BMD has been compared with DXA T-score for BMD in a chronic kidney disease, peritoneal dialysis cohort (*n* = 41) [39], which are liable to artefact via the accumulation of vascular calcification: no significant differences were observed between the BMD T-scores measured through DXA or REMS at the femur, whereas at lumbar spine the anteroposterior DXA mean T-score (affected by calcification artifacts) was significantly higher than both the lateral DXA (more reliable because not affected by calcifications) and the REMS measurements (*p* < 0.01 vs. both).

Aromatase inhibitors (AI), used in consort with surgery and radiotherapy, are a key tool in the treatment of breast cancer, but are complicated by the adverse effect of reduced BMD. Repeated REMS measurements have captured the reduction in BMD, caused by the commencement of AI and the increase in BMD which follows treatment of these patients with denosumab [17]. REMS effectiveness has also been verified in further single-centre studies of patients with anorexia nervosa or with rare diseases such as osteogenesis imperfecta or acromegaly [40–42].

## Deployment and Future Perspectives

The published evidence analysed in the previous sections of this paper have already led to the inclusion of REMS technology in the Italian Health Ministry Guidelines on “Diagnosis, Risk Stratification and Continuity of Care of Fragility Fractures” as a diagnostic innovation capable of addressing unmet clinical needs such as: continuity of care (even in the context of a patient’s home), diagnostic appropriateness, improvement of osteoporosis diagnosis and fracture prevention in clinical practice, short-term bone and therapeutic monitoring to guarantee adherence to therapy.

This represents a personalised approach to bone health assessment and monitoring, characterised by reliable applicability throughout the lifecourse, from younger to older subjects, thanks to high accessibility (due to portability) and the absence of ionising radiation, combined with automatic avoidance of artefacts and availability of additional information with respect to DXA, such as the Fragility Score assessment and the related 5-year fracture risk.

In terms of clinical practice, REMS also has specific scope for deployment in the context of secondary fracture prevention and could fit well within the models of Fracture Liaison Services or Orthogeriatric service. The portable nature of REMS allows it to be used for frail inpatients who might find it difficult to position themselves effectively (and comfortably) for DXA scans of the spine or hip. Indeed, REMS can be performed at the bedside, allowing a rapid review of BMD in the immediate post-fracture period. This might facilitate urgent, BMD-informed decision regarding optimal orthopaedic intervention, as well as risk stratification informing prescription of anti-osteoporosis (anti-resorptive or bone forming agents). A further critical role might be the assessment of BMD during initial presentation to the Emergency Department with a fracture, facilitating more efficient Fracture Liaison Service approaches, addressing a key gap in fracture prevention [43].

In primary prevention of osteoporotic fractures, the non-ionising radiation advantage of REMS allows radiation sensitive populations to receive BMD screening (including pregnant women) but also repeated, regular monitoring of BMD which could be used to track therapeutic response and may have incidental advantages of improving medication adherence and persistence. Given the portability of REMS there is the option to include it in a domiciliary osteoporosis model of care, for those unable to leave their home.

There may be financial benefits to the use of REMS compared to current screening with DXA. A health economic analysis based in the Italian National Health Service reported costs to healthcare professionals of €31.9 for REMS (€48.8 for DXA), costs of testing of €45.1 for REMS (€68.2 for DXA) and thus and overall mean saving (at a health service level) of €40 million per million of patients [44]. This did not include one-off costs which were estimated at €357.4 for REMS training against €1,169.0 for DXA training, and the costs of device acquisition which was estimated at €32,833 for REMS and €45,000 for DXA, which should increase the total saving associated with REMS use.

Further ongoing developments of REMS, besides the application to paediatric patients and to additional anatomical sites, are addressed to the investigation of muscles. Indeed, muscle health is key to advantageous musculoskeletal ageing and a recent pilot study has used REMS to assess muscle strength and further work is planned in this area [45]. The study investigated the relationship between handgrip strength and a novel REMS parameter derived from ultrasound scans of the forearm. REMS acquisitions were performed in two study groups, healthy subjects (*n* = 30) and individuals affected by sarcopenia (*n* = 28), and the novel parameter dedicated to muscle strength estimation was highly correlated with the handgrip measurements in the overall population (*n* = 58, *r* = 0.95, *p* < 0.0001).

## GRADE methodology

In October 2023, The European Society for Clinical and Economic Aspects of Osteoporosis, Osteoarthritis and Musculoskeletal Diseases (ESCEO) convened a working group including clinicians (rheumatologists, endocrinologists, orthopaedic surgeons, gynaecologists, rehabilitation specialists), epidemiologists and public health experts. At the meeting, the latest evidence regarding REMS was reviewed and was synthesised with expert opinion to inform a GRADE (Grading of Recommendations, Assessment, Development, and Evaluations) [46] deployment of REMS in clinical practice.

The GRADE process involved expert members of the working group (*n* = 19 grading a list of statements with a level of agreement (‘agree’, ‘disagree’) and a strength of recommendation (‘recommended’ or ‘not recommended’, rated ‘strong’ to ‘weak’ depending on the extent with which the member agreed with the statement). Members were allowed to choose the most appropriate category and there was a single round of voting.

## GRADE recommendations

The results of the GRADE assessment are shown in supplementary Table 1.

The following recommendations received a grading of “strongly in favour”:

- REMS is a non-ionising diagnostic technology, which informs osteoporosis diagnosis at femur and spine sites.
- REMS can contribute to identification of patients at high risk of fragility fractures.
- REMS can facilitate continuity of care for subjects whose limited mobility precludes DXA scanning.
- Given the lack of ionising radiation, REMS may be used to assess bone health in pregnant women.
- REMS can usefully contribute to optimised post-fracture management of frail patients.

The following recommendations received a grading of “weakly in favour”:

- REMS Fragility Score predicts risk of incident fracture.
- REMS may be used for short-term monitoring of bone health.
- REMS appears less influenced by bone artefacts than DXA (calcifications, osteophytes, prosthesis etc…).

## Conclusions

The current evidence base supports the use of REMS in osteoporosis management as a more widely accessible alternative to DXA for axial BMD measurement. Owing to the lack of ionising radiation, REMS can be safely used in sensitive populations (pregnancy and the young) and for the implementation of dedicated osteoporosis prevention programs, but also allows personalised lifelong monitoring of bone health and therapeutic follow-up applications, thanks to the reported precision values. Furthermore, the portability of the device means that it can be used in contexts where DXA is unavailable, including on hospital wards, in emergency departments, primary care, at home or in low resource settings, and its ability to automatically adjust of degenerative artifacts places it above DXA in relevant cases (e.g. patients heavily affected by osteoporosis of the spine). REMS has also demonstrated efficacy in incident fracture prediction through the use of the Fragility Score, a BMD-independent measure of skeletal fragility (i.e., bone quality). Assessment of REMS technology in further large diverse prospective cohorts will help refine both the specific quantification of fracture risk, and its role in relation to DXA and fracture risk assessment tools such as the FRAX®. In conclusion, REMS offers accessible and safe assessment of BMD, in particular facilitating efficient osteoporosis management in situations where DXA is unavailable or inaccessible. The current evidence base suggests that consideration might now be given to the relative positioning of REMS, as either an adjunct or alternative to DXA, in clinical pathways.

### Electronic supplementary material

Below is the link to the electronic supplementary material.

Supplementary Material 1

## Data Availability

No datasets were generated or analysed during the current study.
