# Supplementary figures and images for: Radiofrequency echographic multi spectrometry (REMS) in the diagnosis and management of osteoporosis: state of the art
Source: Aging Clin Exp Res. 2024 Jun 21;36(1):135. doi: 10.1007/s40520-024-02784-w (PMC11192661; doi:10.1007/s40520-024-02784-w)

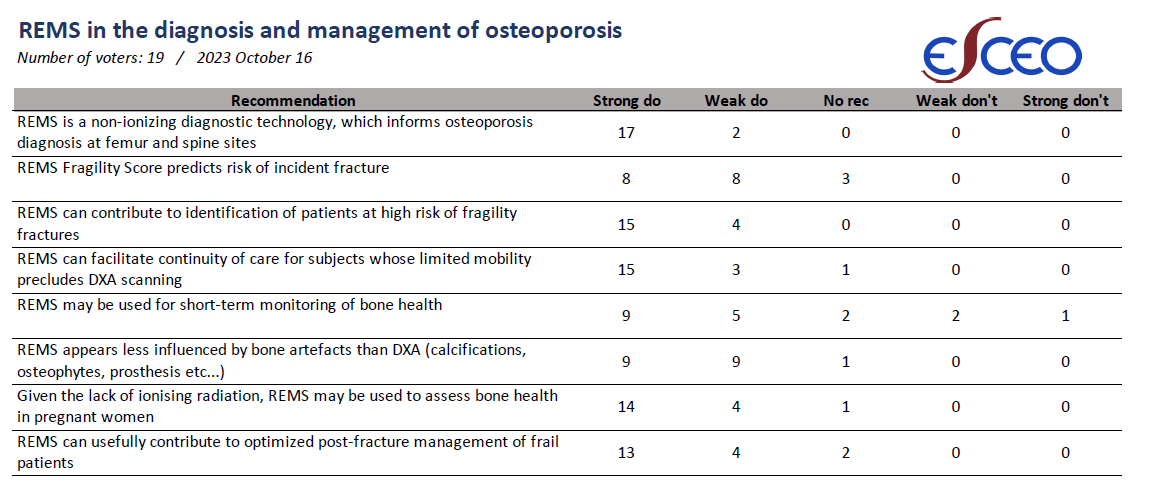

Supplement: Supplementary file 1 — Supplementary Material 1 [file 40520_2024_2784_MOESM1_ESM.docx]
